# The effectiveness of control measures during the 2022 COVID-19 outbreak in Shanghai, China

**DOI:** 10.1371/journal.pone.0285937

**Published:** 2023-05-18

**Authors:** Liangjian Hu, Meisong Shi, Meili Li, Junling Ma

**Affiliations:** 1 College of Science, Donghua University, Shanghai, 201620, China; 2 Department of Mathematics and Statistics, University of Victoria, Victoria, BC, Canada; Shanxi University, CHINA

## Abstract

**Background:**

In March 2022, the Omicron variant of SARS-CoV-2 spread rapidly in Shanghai, China. The city adopted strict non-pharmacological intervention (NPI) measures, including lockdown (implemented on March 28 in Pudong and April 1 in Puxi) and blanket PCR testing (April 4). This study aims to understand the effect of these measures.

**Methods:**

We tabulated daily case counts from official reports and fitted a two-patch stochastic SEIR model to the data for the period of March 19 to April 21. This model considered two regions in Shanghai, namely Pudong and Puxi, as the implementation of control measures in Shanghai was carried out on different dates in these regions. We verified our fitting results using the data from April 22 to June 26. Finally, we applied the point estimate of parameter values to simulate our model while varying the dates of control measure implementation, and studied the effectiveness of the control measures.

**Results:**

Our point estimate for the parameter values yields expected case counts that agree well the data for both the periods from March 19 to April 21 and from April 22 to June 26. Lockdown did not significantly reduce the intra-region transmission rates. Only about 21% cases were reported. The underlying basic reproduction number *R*_0_ was 1.7, and the control reproduction number with both lockdown and blanket PCR testing was 1.3. If both measures were implemented on March 19, only about 5.9% infections would be prevented.

**Conclusions:**

Through our analysis, we found that NPI measures implemented in Shanghai were not sufficient to reduce the reproduction number to below unity. Thus, earlier intervention only has limited effect on reducing cases. The outbreak dies out because of only 27% of the population were active in disease transmission, possibly due to a combination of vaccination and lockdown.

## Background

In March 2022, a rapid increase of COVID-19 cases of the Omicron variant was observed in Shanghai, China. The outbreak lasted until early July, causing huge economic losses [1]. Shanghai implemented strict non-pharmaceutical intervention (NPI) measures, including city lockdown and blanket Polymerase Chain Reaction (PCR) testing, from March 28 to June 1 [2]. Since its first report by South Africa in November 2021, Omicron has rapidly become a strain with global dominance due to its high transmissibility and immune escape [3,4]. Although China’s containment policy was effective in responding to the pre-Omicron outbreak, its effectiveness against Omicron is uncertain. In this study, we aim to estimate the effectiveness of the NPI measures in Shanghai.

The 2022 Shanghai Omicron outbreak has been extensively studied. For example, Chen et al. [5] provided a comprehensive overview of the temporal development and spatial distribution of this outbreak, and concluded that the targeted interventions in Shanghai in March 2022 will not stop the spread of Omicron and that the implementation of a strict and long-term lockdown will be successful in containing the outbreak. Xie et al. [6] developed a Lévy-PSO algorithm and applied it to a fractional-order SEIAR model and calibrated the model to the number of people infected with COVID-19 in Shanghai. Lou et al. [7] constructed a SEPASHRD model considering the age structure and vaccination stratification and learned that precise prevention and control in Shanghai at the beginning could not effectively respond to Omicron and that appropriate measures at the optimal time of intervention were necessary to effectively curb the epidemic growth. Liu et al. [8] constructed a model based on SEIR model and combined it with a logistic growth model for numerical simulation. The final parameters obtained were able to match the actual number of cases better.

These studies did not consider the regional nature of the implementation of control measures in Shanghai, and assumed that the population in Shanghai remained randomly mixed during outbreak. In this study, we use a two-patch SEIR model to incorporate the regional implementation of NPI measures in Pudong and Puxi and calibrate the model to daily case counts in these two regions. We then study the basic and control reproduction numbers, and estimate the effect of control measures in Shanghai.

## Materials

The outbreak in Shanghai started on March 1 and ended on May 31, 2022 [2]. Official reports of daily case counts were published for each district in Shanghai from March 19, 2022. We tabulated the published daily counts of confirmed cases and asymptomatic cases for each region in Shanghai from March 19 to June 26, 2022 [1]. An asymptomatic case was classified as a PCR-confirmed individual if they had no self-perceived and clinically recognizable symptoms such as fever, cough, sore throat and no radiological evidence of pneumonia [5]. In this study, we use the sum of confirmed and asymptomatic cases from March 19 to June 26 as the daily case counts.

In response to this outbreak in Shanghai in early March, the Shanghai government took a series of NPI measures, such as city lockdown, blanket PCR screening, case isolation, tracing of close contacts (requires quarantine in separate facilities) and the secondary contacts. When Omicron cases first emerged in early March, Shanghai adopted a precise-prevention-and-control policy that allowed people to live a normal productive life. From March 16 to 27, Shanghai divided the street blocks into high-risk and low-risk zones, and during this period, the city conducted 1–2 rounds of blanket PCR screening in the high-risk zones [5].

Since March 28, control measures such as lockdown and blanket testing were implemented regionally in Shanghai, first to the east bank of Huangpu River, and then to the west on April 1. In this paper, we call the east bank of Shanghai Pudong (including all Pudong New District, Fengxian District, Jinshan District, Chongming District, the Pujin Street and the Township of Pujiang in Minhang District, and the Townships of Xinbang, Shihutang, Mao Port and Ye Xie in Songjiang District), and call the remaining districts Puxi.

**Fig 1:**
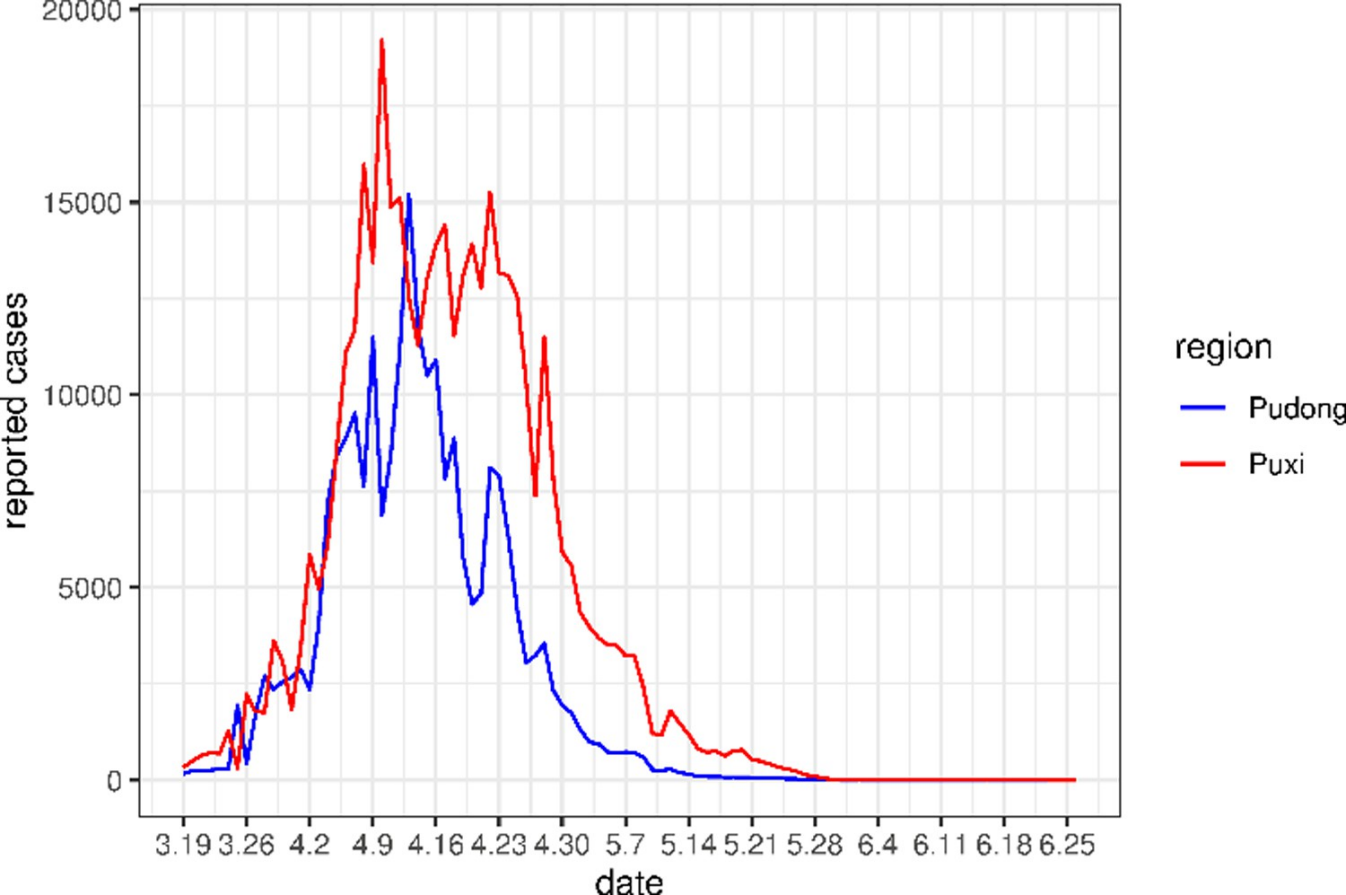
The number of daily reported cases in Pudong and Puxi.

Pudong entered a phased lockdown starting at 5:00AM on March 28, and PCR screening was gradually carried out in the region. Starting at 3:00AM on April 1, lockdown and gradual PCR screening was implemented in Puxi [9]. On April 4, the city implemented city-wise blanket PCR screening. According to the seventh national census data, the resident population of Shanghai is 24,870,895 [10]. Among them, the resident population in Pudong is *N*_*pd*_ = 8,283,100 and in Puxi is *N*_*px*_ = 16,588,000. The number of daily reported cases in Pudong and Puxi are plotted in Fig 1.

Since April 4, Shanghai began and maintained a high frequency of blanket PCR testing [11].

## Methods

### Model

The classic SEIR model is widely used in epidemiological modeling. It divides the population into four classes, susceptible (S), exposed (E), infected (I) and recovered (R). The model has been widely used to study the outbreak of COVID-19 and other infectious diseases [12–18]. Because the control measures in Shanghai were implemented regionally in Pudong and Puxi (see Section Materials for details), we develop a stochastic two-patch SEIR model to describe the outbreak in Shanghai. Though asymptomatic transmission may be an important factor of COVID-19 dynamics, we ignore it in our model to reduce the number of model parameters that needs to be fitted. We assumed that a fraction *τ* of the infectious individuals was detected and notified, and a fraction *γ* of the infectious individuals was self-recovered and not notified.

#### The base model: From March 19 to March 27 (before the lockdown)

We assume that the intra-region transmission rate within Pudong and Puxi are both *β*_11_, and inter-region transmission rate is *β*_12_ in this stage.


$$Incidences\;in\;Pudong:X_{pd_{t}}\sim Poission((\beta_{11}I_{pd_{t-1}}+\beta_{12}I_{px_{t-1}})S_{pd_{t-1}}/N_{pd})$$ (1)

$$Incidences\;in\;Puxi:X_{px_{t}}\sim Poission((\beta_{11}I_{px_{t-1}}+\beta_{12}I_{pd_{t-1}})S_{px_{t-1}}/N_{px})$$ (2)

$$symptom\;onsets\;in\;Pudong:Y_{pd_{t}}\sim Binomial(E_{pd_{t-1}},\sigma )$$ (3)

$$Symptom\;onsets\;in\;Puxi:\;Y_{px_{t}}\sim Binomial\left(E_{px_{t-1}}\;,\;\sigma \right)$$ (4)

$$Recovery\;in\;Pudong:\;Z_{Pd_{t}}\sim Binomial\left(I_{pd_{t-1}}\;,\;\gamma \right)$$ (5)

$$Recovery\;in\;Puxi:\;Z_{px_{t}}\sim Binomial\left(I_{px_{t-1}},\;\gamma \right)$$ (6)

$$Tested\;in\;Pudong:\;P_{pd_{t}}\sim Binomial\left(I_{pd_{t-1}},\;\tau_{1}\right)$$ (7)

$$Tested\;in\;Puxi:\;P_{px_{t}}\sim Binomial\left(I_{px_{t-1}},\;\tau_{1}\right)$$ (8)

$$Dynamics:\left\{\begin{array}{c} S_{pd_{t}}=S_{pd_{t-1}}-X_{pd_{t}}\\ S_{px_{t}}=S_{px_{t-1}}-X_{px_{t}}\\ E_{pd_{t}}=E_{pd_{t-1}}+X_{pd_{t}}-Y_{pd_{t}}\\ E_{px_{t}}=E_{px_{t-1}}+X_{px_{t}}-Y_{px_{t}}\\ I_{pd_{t}}=I_{pd_{t-1}}+Y_{pd_{t}}-Z_{pd_{t}}-P_{pd_{t}}\\ I_{px_{t}}=I_{px_{t-1}}+Y_{px_{t}}-Z_{px_{t}}-P_{px_{t}} \end{array}\right.$$ (9)

In general, the mean field approximation of this stochastic model is

$$\begin{array}{l} S_{pd}^{'}=-\left(B_{11}I_{pd}+B_{12}I_{px}\right)S_{pd}/N_{pd}\\ S_{px}^{'}=-\left(B_{21}I_{pd}+B_{22}I_{px}\right)S_{px}/N_{px}\\ E_{pd}^{'}=\left(B_{11}S_{pd}I_{pd}/N_{pd}+B_{12}S_{pd}I_{px}/N_{pd}\right)-\tilde{\sigma }E_{pd}\\ E_{px}^{'}=\left(B_{21}S_{px}I_{pd}/N_{px}+B_{22}S_{px}I_{px}/N_{px}\right)-\tilde{\sigma }E_{px}\\ I_{pd}^{'}=\tilde{\sigma }E_{pd}-\tilde{\gamma }I_{pd}-\tilde{\tau }I_{pd}\\ I_{px}^{'}=\tilde{\sigma }E_{px}-\tilde{\gamma }I_{px}-\tilde{\tau }I_{px}\\ R_{pd}^{'}=\tilde{\gamma }I_{pd}\\ R_{px}^{'}=\tilde{\gamma }I_{px}\\ T_{pd}^{'}=\tilde{\tau }I_{pd} \end{array}$$

$$T_{px}^{'}=\tilde{\tau }I_{px}$$ (10)

where *B*_11_ represents the intra-region transmission rate in Pudong, *B*_12_ represents the inter-region transmission rate from Puxi to Pudong, *B*_21_ represents the inter-region transmission rate from Pudong to Puxi, *B*_22_ represents the intra-region transmission rate in Puxi. The probabilities in the stochastic model are related to the rates in (10) as

$$\sigma =1-e^{-\tilde{\sigma }},\;\gamma =1-e^{-\tilde{\gamma }},\;\tau =1-e^{-\tilde{\tau }}.$$

Then in the initial stage, *B*_11_ = *B*_22_ = *β*_11_, *B*_12_ = *B*_21_ = *β*_12_. Note that we assume that the population sizes are constants in this period, because births and deaths (including disease induced deaths) are negligible during this period in Shanghai.

#### Second stage: From March 28 to March 31 (Pudong lockdown)

In this stage, we assume that the lockdown is effective in preventing any travel between Pudong and Puxi. Thus, the inter-region transmission rate *B*_12_ = *B*_21_ = *β*_12_ = 0. In addition, the intra-region transmission in Pudong is reduced to *B*_11_ = *β*_21_, and a fraction 1—*q* of the susceptible population is removed from disease transmission. Since the number of infected individuals is a small fraction of the total population, $S_{pd_{t-1}}\approx N_{pd}$, and thus the remaining susceptible population is $S_{pd_{t-1}}-\left(1-q\right)N_{pd}$, and the remaining population is approximately *qN*_*pd*_. So,

$$X_{pd_{t}}\sim Poission\left(\beta_{21}I_{pd_{t-1}}\left[S_{pd_{t-1}}-N_{pd}\left(1-q\right)\right]/\left(qN_{pd}\right)\right)$$ (11)

$$X_{px_{t}}\sim Poission\left(\beta_{11}I_{px_{t-1}}S_{px_{t-1}}/N_{px}\right)$$ (12)

The symptom onsets, recovery, tested patients and the dynamics follow (3)—(9). Note that here we made an implicit assumption that $S_{pd_{t-1}}>N_{pd}\left(1-q\right)$. This may not be true for some extreme parameter values. However, since the total number of infected individuals is a small fraction of the total population in both Pudong and Puxi, this assumption is realistic, as is demonstrated by our fitting result.

#### Third stage: From April 1 to April 3 (lockdown in both Puxi and Pudong)

In this stage, city lockdown is implemented in Puxi as well, then *B*_22_ = *β*_21_. Thus,

$$X_{px_{t}}\sim Poission\left(\beta_{21}I_{px_{t-1}}\left[S_{px_{t-1}}-N_{px}\left(1-q\right)\right]/\left(qN_{px}\right)\right)$$ (13)

The other equations remain unchanged from the previous stage.

#### Fourth stage model: From April 4 to April 21 (blanket PCR testing)

In this stage, blanket testing is implemented in both regions. We assume that the testing rate is increases to *τ*_2_ > *τ*_1_.


$$P_{pd_{t}}\sim Binomial\left(I_{pd_{t-1}},\;\tau_{2}\right)$$ (14)

$$P_{px_{t}}\sim Binomial\left(I_{px_{t-1}},\;\tau_{2}\right)$$ (15)

The other equations remain unchanged from the previous stage.

### Basic reproduction number

The basic reproduction number *R*_0_ is a key parameter in epidemiology that determines whether an outbreak will occur [19]. It is the average number of secondary infections caused by a typical infected individual in a fully susceptible population. If *R*_0_ > 1, the infectious disease will break out, otherwise it will be eliminated.

Use the next generation matrix method [20], the basic reproduction number for our model (10) can be calculated as

$$R_{0}=\frac{B_{11}+B_{22}+\sqrt{{\left(B_{11}-B_{22}\right)}^{2}+4B_{12}B_{21}}}{2\left(\tilde{\tau }+\tilde{\gamma }\right)}.$$

The basic reproduction number during the period prior to the lockdown (March 19 to March 27) was determined to be

$$R_{0}=\frac{\beta_{11}+\beta_{12}}{{\tilde{\tau }}_{1}+\tilde{\gamma }},$$

since *B*_11_ = *B*_22_ = *β*_11_ and *B*_12_ = *B*_21_ = *β*_12_ at this stage. After the lockdown in Pudong (March 28 to March 31), the control reproduction number was

$$R_{c}=\frac{\text{max}(\beta_{11},\beta_{21})}{{\tilde{\tau }}_{1}+\tilde{\gamma }},$$

because we assume *B*_11_ = *β*_21_, *B*_22_ = *β*_11_ and *B*_12_ = *B*_21_ = *β*_12_ = 0 in this period. After the lockdown in Puxi (April 1 to April 3), the control reproduction number was calculated to be

$$R_{c}=\frac{\beta_{21}}{{\tilde{\tau }}_{1}+\tilde{\gamma }},$$

since *B*_11_ = *B*_22_ = *β*_21_ and *B*_12_ = *B*_21_ = *β*_12_ = 0 in this period. Moreover, after the implementation of blanket PCR testing (April 4 to April 21), the control reproduction number changed to

$$R_{c}=\frac{\beta_{21}}{{\tilde{\tau }}_{2}+\tilde{\gamma }}.$$

The effective reproduction numbers are smaller than the control values because a fraction of the population had been infected when the control measures were implemented.

### Parameter estimation

The population sizes in both regions are given in Table 1 [10].

**Table 1:** The population sizes.

Variable	Meaning	data
***N***_***pd***_	Resident Population in Pudong	8,283,100
***N***_***px***_	Resident Population in Puxi	16,588,000

The model parameters *β*_11_, *β*_12_, *β*_21_, *ϭ*, *γ*, *τ*_1_, *τ*_2_, *q* are estimated using Markov Chain Monte Carlo (MCMC) via the R package R2jags. The parameters and the prior distributions are listed in Table 2. The model is fit to the daily case data, which corresponds to $P_{pd_{t}}$ and $P_{px_{t}}$ for the time *t* from March 19 to April 21. Because our model does not distinguish symptomatic and asymptomatic infections, the daily case counts are the sum of asymptomatic and symptomatic cases reported every day.

**Table 2:** Model parameter details.

Parameter	Meaning	Prior distribution
***β***_**11**_	intra-region transmission rate before lockdown	uniform distribution from 0–10
***β***_**12**_	inter-region transmission rate before lockdown	uniform distribution from 0–5
***β***_**21**_	intra-region transmission rate after lockdown	uniform distribution from 0–10
***σ***	the rate that latent individuals leaving the class	uniform distribution from 0.1–1
***ϒ***	the recovery rate	normal distribution with mean 5, sd 1
***τ***_**1**_	the proportion detected before full nucleic acid testing	uniform distribution from 0–1
***τ***_2_	the proportion detected after full nucleic acid testing	uniform distribution from *τ*_1_−1
***q***	the proportion of susceptible individuals	uniform distribution from 0–1

### Model validation

We numerically solve the mean-field model (10) using the initial conditions and parameter values from our point estimate to produce the expected number of cases in Pudong and Puxi. The parameter values are time dependent analogously to the stochastic model. We compare the expected cases in these regions with the data from April 22 to June 26.

## Results

### Fitting results

Table 3 shows the fitting results for each parameter, including the mean values and 95% confidence intervals. The intra-region transmission rate *β*_21_ after lockdown is not statistically significantly different from *β*_11_. Yet there is a significant increase in the testing rate *τ*_2_ after blanket PCR testing.

**Table 3:** Estimated parameter values (95% confidence intervals in parentheses).

Parameter	Estimates
***β***_11_	0.99 (0.90, 1.06)
***β***_12_	0.24 (0.22, 0.25)
***β***_21_	1.03 (0.93, 1.10)
***σ***	1.00 (1.00, 1.00)
***ϒ***	0.63 (0.56, 0.69)
***τ***_**1**_	0.10 (0.09, 0.10)
***τ***_2_	0.16 (0.15, 0.18)
***q***	0.27 (0.26, 0.28)

We use SEIR model to simulate the epidemic. Using the point estimates, we obtain the basic regeneration number *R*_0_ of the model by calculating the Jacobian matrix at the disease-free equilibrium point.

Using the point estimated values, the basic reproduction number is 1.7 before the lockdown (March 19 to March 27), the control reproduction number is 1.4 after the lockdown in Pudong (March 28 to March 31), 1.4 after the lockdown in Puxi (April 1 to April 3), and 1.3 after the implementation of blanket testing (April 4 to April 21).

**Fig 2:**
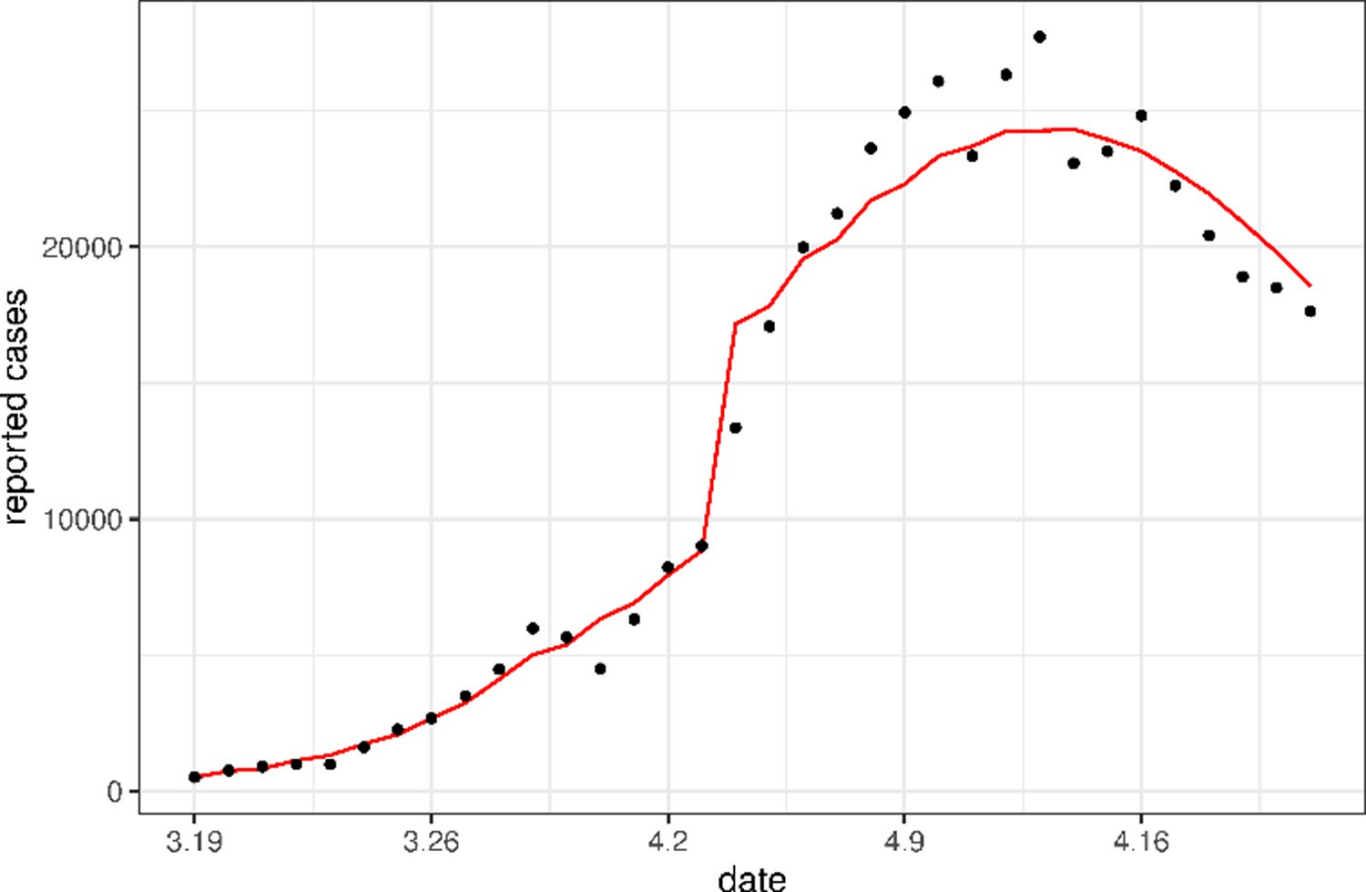
Comparison of the predicted daily number of new cases (red solid line) with the real data (circle scatter). The predicted cases are obtained by calculating the mean dynamics of the stochastic models (1)-(9) and (11)-(15) with the point estimates of the parameters.

**Fig 3:**
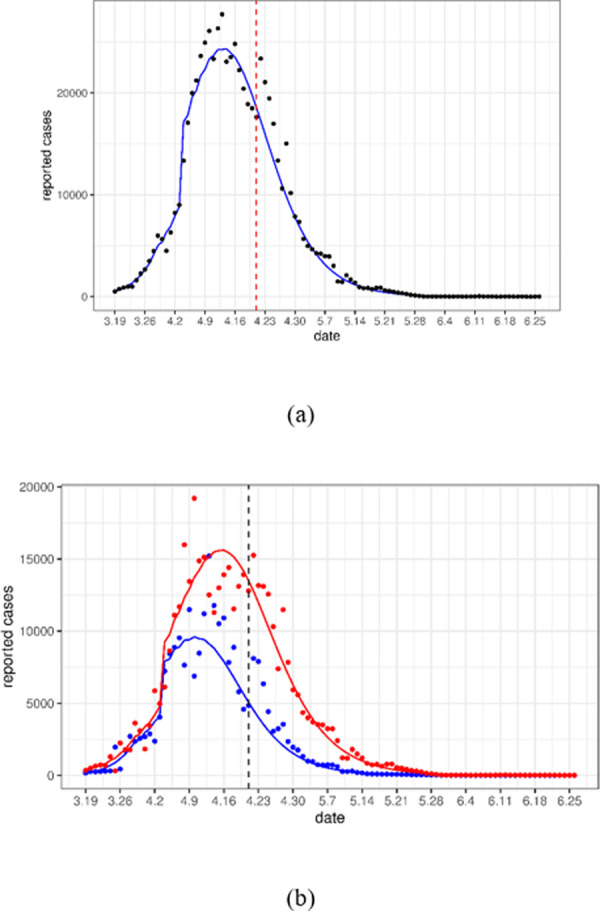
Comparison of the predicted daily number of new cases (red solid line) with the real data (circle scatter) beyond the time period for fitting our model (before the dashed line). The predicted cases are obtained by calculating the mean dynamics of the stochastic models (1)-(9) and (11)-(15) with the point estimates of the parameters. Panel (a) shows the total cases, and panel (b) shows the cases in Pudong (red) and Puxi (blue).

Using the point estimates (the mean) of the parameter values, we plotted the solutions of the mean-field model (10) as the expected daily case counts in Fig 2. These expected curves well matched the observed cases very well. In Fig 3, we plotted the curve of the number of cases per day for a period of 100 days from March 19 to June 26 using the fitted values obtained in Table 3. This figure shows that our model matched the reported cases very well even beyond the fitted periods (i.e., after April 21).

### Optimal control

From March 19 to June 26, a cumulative total of 647,607 cases were reported in Shanghai. The cumulative number of infections (tested cases plus self-recovered cases) was calculated by our model to be 3,157,840 cases, i.e., only 21% of cases were reported.

**Fig 4:**
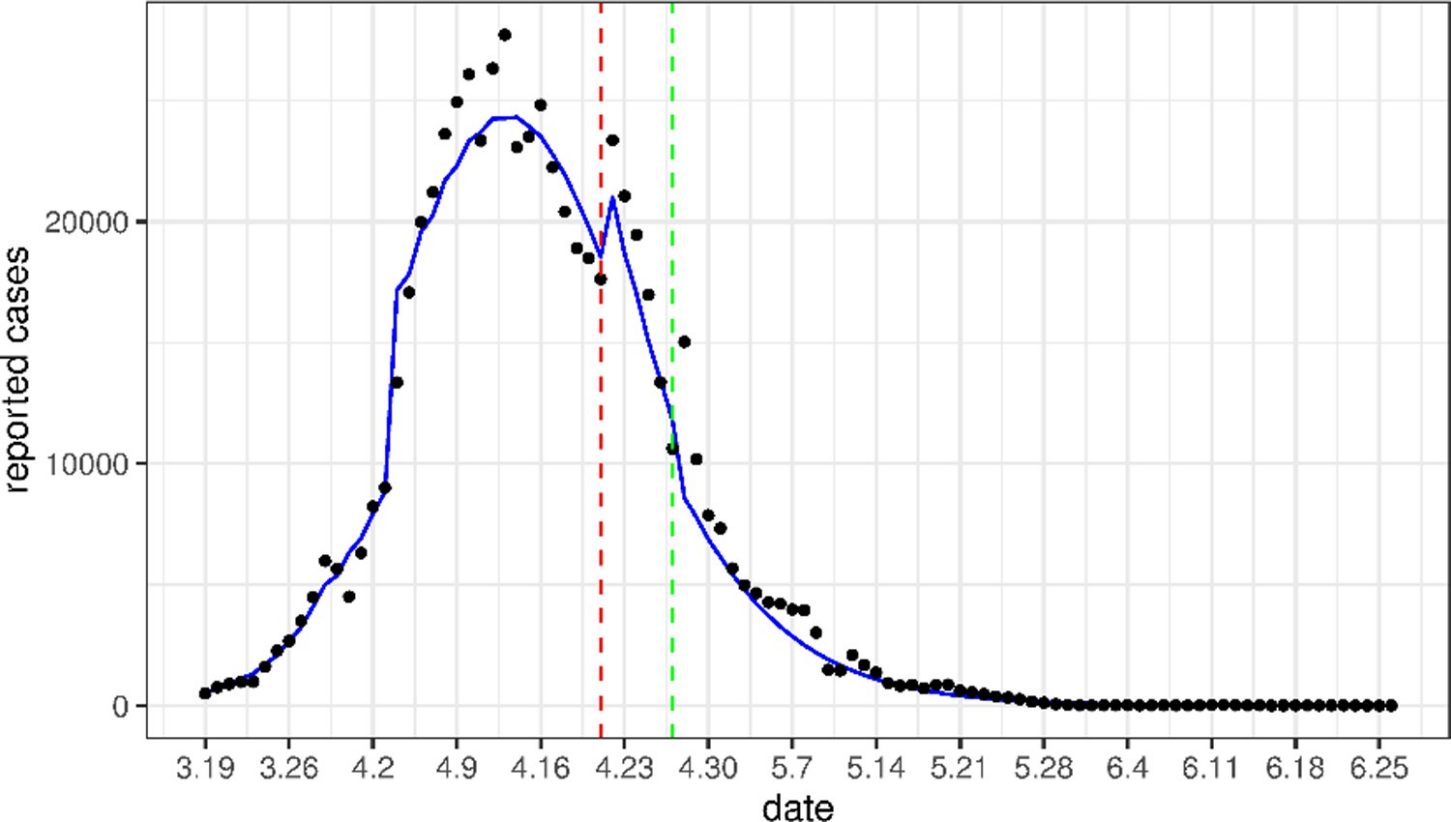
Comparison of the daily number of new cases (blue solid line) obtained by applying the parameter fitting results with the real data (circle scatter). Unlike Fig 3, here the detection rate increased to 0.198 from April 22 to April 27, to match the social clearance campaign in this period.

From Fig 3, we can see that the number of cases from April 22 to April 27 is higher than we predicted, which is because Shanghai launched a general social clearance campaign during this period, resulting in an increase in detection rates [21]. Assuming that the detection rate *τ* increases to 0.198 during this period, and then decreases to *τ*_2_ after April 27, the predicted case counts are plotted in Fig 4. The predicted value remains in line with the actual value even after April 27. This indicates that the increase testing during these 6 days has no significant effect on the disease dynamics after this period.

**Fig 5:**
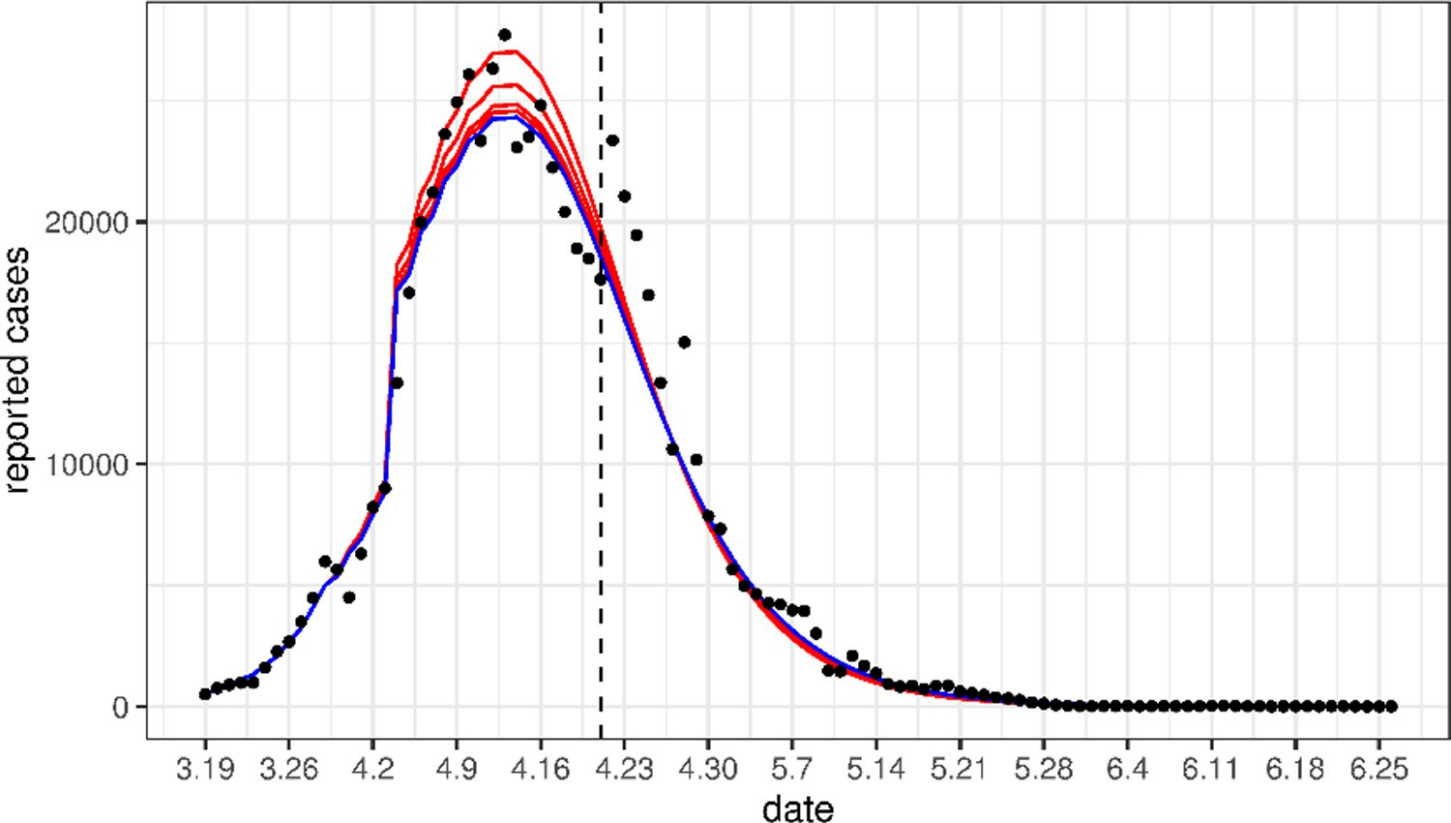
Comparison of the predicted daily cases for a range of transmission rates 0.24*ε* after lockdown (where 0.24 is the point estimate of the inter-region transmission rate *β*_12_ before lockdown), for *ε* = 0.01, 0.02, 0.05, 0.1 (red solid line, higher curves correspond to higher *ε* values) and *ε* = 0 (blue solid line) with the real data (circle scatter).

To study the sensitivity of our result on the assumption that the inter-region transmission rate *β*_12_ = 0 after the lockdown, we plot in Fig 5 the predicted daily cases for a range of transmission rates 0.24*ε* after lockdown (where 0.24 is the point estimate of the inter-region transmission rate *β*_12_ before lockdown), for *ε* = 0.01, 0.02, 0.05, and 0.1. This figure shows that our results are not sensitive to this assumption.

**Fig 6:**
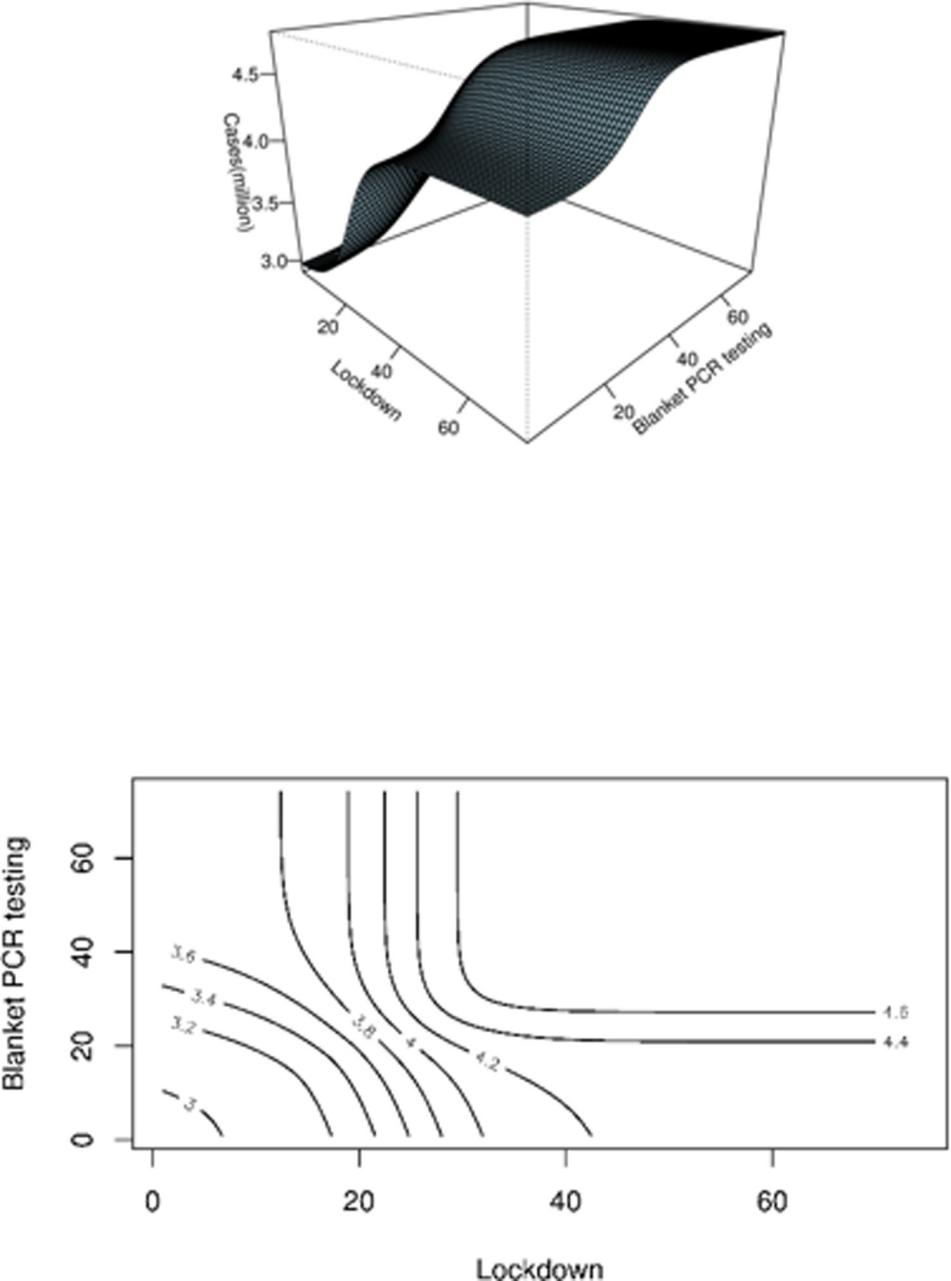
Three-dimensional plot and the contour plot of the total (including asymptomatic) cases as a function of the starting dates for lockdown and blanket testing.

Fig 6 shows the expected total (including unreported) cases for various start dates for the lockdown and blanket testing, starting from March 19 (i.e., the first day in our model). The cumulative number of infections would be reduced to 2,975,794 (Fig 6), i.e., about 185,046 (5.7%) fewer cases, if both control measures were implemented from March 19. If the inter-region transmission rate is 0.24*ε* > 0 after the lockdown, for the same *ε* values as in Fig 5, the change in the results of Fig 6 is less than 6.6%.

## Conclusions

In this study, we fitted a stochastic two-patch SEIR model to the daily cases counts in Shanghai for the period March 19 to April 21, to incorporate the implementations of control measures on the two regions of Shanghai (Pudong and Puxi that are separated by Huangpu River).

Our results suggest that the implementation of blanket PCR testing significantly increased the testing rate. On the other hand, the lockdown did not significantly reduce the transmission rate in each region. As a result, the intra-region transmission for a typical patient remained the same before and after lockdown. However, the lockdown significantly reduced the exposure of susceptible individuals by preventing 73% susceptible from disease transmission. This caused the epidemic to quickly deplete the available susceptible population and peak. However, a high vaccine coverage in Shanghai (85% by March 1, 2022 [22]) could also have contributed to the reduction of exposure.

Our model also predicts that, even with both lockdown and blanket testing implemented, the control reproduction number is 1.3 > 1, which implies the control measures were not sufficient to curtail the disease spread. The effect of city lockdown has been stopping the inter-region transmission between Pudong and Puxi. The subsequent decrease in transmission rate was due to the depletion of the susceptible population, which is because only 27% of the population were available for disease transmission. This also implies that a larger fraction of the population is infected. Our results suggest that only 21% of the cases were reported. In addition, because the control reproduction number is 1.3 with both control measures, even if they were implemented as early as possible on March 19, about 185 thousand cases would have avoided infection. This corresponds to a reduction of only 39 thousand (or 5.7%) reported cases.

## Supporting information

S1 File The R code for model fitting and case count data can be found at: https://github.com/junlingm/Shanghai-COVID-19-Mar-June-2022.git. (CSV)
